# Special Issue on the Latest Research on Flavor Components and Sensory Properties of Food during Processing and Storage

**DOI:** 10.3390/foods12203761

**Published:** 2023-10-13

**Authors:** Magdalena Gantner, Eliza Kostyra

**Affiliations:** Department of Functional and Organic Food, Institute of Human Nutrition Sciences, Warsaw University of Life Sciences, Nowoursynowska Str. 159c, 02-776 Warsaw, Poland; eliza_kostyra@sggw.edu.pl

Due to their chemical composition and physico-chemical properties, most food products are susceptible to biochemical, microbiological, physical and chemical deterioration. In order to extend their shelf-life, enable their transport and storage, and ultimately ensure their safe consumption, special technological treatments are required to prevent or minimise the occurrence of these adverse factors.

Food processing includes various methods of transforming raw ingredients into food products, such as cooking, canning, freezing, drying, fermentation, irradiation, or the use of high-pressure technology. Several food preservation techniques are employed to extend the shelf life of food products, such as refrigeration, drying, salting, sweetening, pickling, and the use of preservatives. Processing and proper storage contribute to the elimination or inhibition of the growth of harmful microorganisms that can cause food poisoning or spoilage.

However, processing and storage can also cause adverse changes and interactions in food ingredients that affect their nutritional value or sensory properties [1]. Some ingredients are transformed into new compounds, while others are lost or inactivated. Sometimes, compounds unfavourable to the health of the consumer are formed. Some chemical changes that occur in food during storage and processing are beneficial, and these include the formation of antioxidants, flavour compounds, pigments and vitamins. Others are detrimental, including the loss of nutrients, formation of toxic compounds, and degradation of texture, colour and aroma [2]. For example, the drying process, on the one hand, has many advantages, resulting in a reduction in weight and volume, which makes it easier to store, package and transport food, as well as providing flavour and aroma, but on the other hand, it has some limitations. In some cases, a significant loss of flavour and aroma is observed after drying, and some functional compounds such as vitamin C, thiamin, protein and lipids are lost due to the application of this process [3]. High temperatures during pasteurisation can damage some vitamins, minerals and beneficial bacteria. Under high-temperature pasteurisation, the concentration of vitamin C is reduced by 20 per cent, and that of soluble calcium and phosphorus is reduced by 5 per cent, while that of thiamine and vitamin B12 is reduced by 10 per cent. In fruit juices, pasteurisation reduces vitamin C and carotene levels [3]. The cooling process delays the growth of microorganisms and inhibits the metabolic activity of intact plant tissues after harvest. It also inhibits the metabolic activity of animal tissues after slaughter and the deterioration of chemical reactions including enzyme-catalysed oxidative browning, lipid oxidation, and chemical changes associated with colour degradation. However, chilling can affect the texture of selected foods and reduce their crispness [3].

Therefore, in order to minimise the negative effects of these processes and obtain food of the highest quality, it is necessary to obtain as much knowledge as possible about the effects of storage and technological treatments on food ingredients. Furthermore, a better understanding of the changes that occur during different forms of processing and storage and their impact on the sensory profile of raw materials and food products can encourage the industry to introduce various innovations. The articles in this Special Issue aim to provide knowledge on important and interesting developments in the understanding of the impact of current processing and storage methods on food quality, with a particular focus on flavour components, sensory properties, and processes.

While agricultural scientists are working to increase the supply to the world’s growing population by increasing yields, the goal of food scientists remains to ensure that the agricultural commodities produced are suited to the nutritional needs of consumers around the world, while remaining sustainable and safe, but also reducing food waste [4].

This Special Issue aims to bring together the latest research on preventing food spoilage, extending shelf life and monitoring storage conditions to produce high-quality food that is completely safe and acceptable to consumers. This Special Issue is intended for food scientists, technologists, food industry professionals, nutritionists, and dieticians.

Recently, many discoveries have been made in the field of food storage, spoilage and shelf life extension. These technologies aim to improve the quality, shelf life and sustainability of various food commodities such as fruit, vegetables, cereals, dairy products, meat, and seafood. Food preservation technologies improve food safety, quality, and sustainability, thereby reducing food waste and increasing the sustainability of the food supply [5]. Therefore, researchers are engaged in a continuous effort to optimise the effectiveness of traditional preservation methods and to develop new ones [6].

Many research topics are being addressed. One of these is the use of nano formulations, modified atmospheres, and methods to prevent food loss and ensure food safety throughout the production and supply chain [6,7,8]. Ordon et al. [9], in their study, found that microbial contamination that can occur in cooked ham comes from the improper cooking of the meat product, inadequate decontamination practices and/or recontamination during packaging, following an increase in the number of microorganisms within the original shelf life. The authors observed that dividers and bags coated with an antimicrobial layer containing *U. tomentosa* extract and ZnO nanoparticles were the best bags for storing finished cooked sliced ham purchased from the local market. Another topic is the monitoring of factors during storage, such as temperature, humidity, ventilation, pest and pathogen control, and packaging and transport, and consequently of the impact of these factors on the quality and shelf life of various food commodities [10,11,12]. The results of a study by Szymczak et al. [13] showed that herring marinated at 2 °C had a higher mass yield but lower non-protein nitrogen (NPN), peptide and free amino acid fractions than that marinated at 7 and 12 °C, and higher temperatures increased the free amino acid content the most and reduced the hardness of the marinated meat as determined via sensory evaluation. The prevailing themes also relate to the development of new technologies and materials to extend the shelf life of fresh and processed foods, such as edible coatings, active packaging, biopreservatives, and natural antioxidants [14,15,16]. As highlighted by Adriani and Handayani [14] in their review article, edible coatings made of natural biopolymers such as lipids, proteins, polysaccharides and composite polymers can be used as alternative food preservatives. They can also be used as carriers of antioxidant and antimicrobial ingredients through the addition of essential oils or the addition of natural ingredients. Food safety risks posed by foodborne pathogens seriously threaten human health and economic stability. Therefore, the identification and characterisation of foodborne pathogens and spoilage-causing microorganisms and the development of rapid and sensitive methods for their detection and control are equally timely topics [17,18,19]. According to Wei and Zhao [18], in recent years, there have been notable developments in the technologies for pathogen detection, especially in the fields of multilocus sequence analysis, clustered regularly interspaced short palindromic repeats (CRISPR), phospholipid fatty acid analysis, and whole-genome sequencing, as potential subtyping methods for improving foodborne pathogen detection.

According to Aladhadh [17], there have been significant recent advances in food preservation methods aimed at extending the shelf life of foods and eliminating spoilage and/or pathogenic microbial groups. Some of the commonly used methods include low-temperature storage (refrigeration and freezing), the use of chemical preservatives (e.g., essential oils and bacteriocins) [20,21,22,23] and vacuum and modified atmosphere packaging [24,25].

Despite the unmistakable benefits of the ever-increasing knowledge of food processing and storage, including improvements in appearance, flavour or aroma, adverse changes often occur, reducing nutritional and technological value due to the appearance of toxic compounds with negative effects on sensory properties.

In this Special Issue, we will focus not only on modern methods, food processing and storage, but also on verifying the impact of these methods on food quality in terms of taste and sensory properties.

Food products are complex multi-component systems consisting of volatile and non-volatile substances. There are many pathways for the biochemical and chemical production of flavour. Some are well known, while others are more speculative [26]. In plant foods, many flavour compounds (or their precursors) are formed via biochemical reactions catalysed by enzymes. Many microbial and enzymatic processes are responsible for the production of hundreds of natural flavour compounds [16]. On the other hand, many changes in aroma and flavour can occur during the storage of products. The cooking process is also integral to the development of flavour in foods. Reactions that lead to enhanced flavour in cooked foods include components such as lipids, sugars, amino acids, carotenoids, and vitamins. The main volatile compounds found in cooked foods are the auto-oxidation products of lipid fatty acids, such as aliphatic aldehydes, ketone alcohols, furans and lactones. Interesting examples from the literature on this subject can be found [27]. It is necessary to understand the chemistry of taste and flavour, especially with regard to fresh and processed foods and novel foods. In addition, flavour chemists have attempted to link the chemical knowledge of flavour compounds to their sensory properties [28]. Another aspect of research is flavour creation, which is seen as a combination of science and art to achieve the desired experiences of taste.

Sensory evaluation is the science of measuring, analysing and interpreting human responses to products perceived via the senses (sight, smell, taste, touch and hearing) [29]. It pertains to sensitivity, precision, accuracy, and the prevention of false alarms [30]. Sensory tests provide valuable information on the human perception of product changes caused by certain ingredients, processing, packaging or shelf life. A traditional sensory methodology is generally subdivided into (1) discriminative tests (which are mainly used to assess significant differences between two or more products), (2) descriptive tests (to characterise differences and similarities between products) and (3) consumer tests (to understand consumers’ hedonic and emotional responses to products) [31]. In addition, new methods have been developed to measure product attributes and consumer responses, such as rapid sensory profiling techniques (e.g., Check-All-That-Apply Questions, Napping, Ideal Profiling) [32]. It is also possible to determine changes in the perception of key sensory and emotional attributes over time as a product is consumed using methods such as the temporal dominance of sensation and temporal dominance of emotion. On the other hand, innovative technologies such as biometric techniques (those measuring facial expression, eye tracking, body temperature, and skin conductivity), contextual environments (virtual and augmented reality), artificial intelligence, and smart sensors (e-nose and e-taste) are currently used in sensory research to better understand human food perception [31]. Researchers are also trying to establish relationships between sensory and instrumental methods.

Researchers and the industry can use a wide range of sensory and consumer methods, as well as innovative technologies, to address a variety of research problems related to the development of new products, the optimisation process, the re-formulation of existing products, the effects of processing and storage on the sensory properties of products, understanding how product attributes affect perception (e.g., exploring food-flavour interactions), and the more in-depth understanding of consumer choices, behaviours, well-being and satisfaction. Sensory techniques are also useful for taste researchers to understand how flavour components contribute to the perceived quality of food.

We invite researchers investigating the impact of current processing and storage methods on food quality, with a particular focus on flavour components and sensory properties, to submit their work to this collection, which aims to cover a wide range of current topics related to these issues.
